# Medical Image Encryption Using Chaotic Mechanisms: A Study †

**DOI:** 10.3390/bioengineering12070734

**Published:** 2025-07-04

**Authors:** Chin-Feng Lin, Yan-Xuan Lin, Shun-Hsyung Chang

**Affiliations:** 1 Department of Electrical Engineering, National Taiwan Ocean University, Keelung 20224, Taiwan; lcf1024@mail.ntou.edu.tw; 2 School of Medicine, Chung Shan Medical University, Taichung 402307, Taiwan; 1201106@live.csmu.edu.tw; 3 Department of Microelectronics Engineering, National Kaohsiung University of Science and Technology, Kaohsiung 81157, Taiwan

**Keywords:** medical image encryption, chaotic map, security evaluation, technical notes, technical features and effectiveness

## Abstract

Medical clinical images have a larger number of bits, and real-time and robust medical encryption systems with a high security level, a large key space, high unpredictability, better bifurcation behavior, low computational complexity, and good encryption outcomes are significant design challenges. Chaotic medical image encryption (MIE) has become an important research area in advanced MIE strategies. Chaotic MIE technology can be used in medical image storage systems, cloud-based medical systems, healthcare systems, telemedicine, mHealth, picture archiving and communication systems, digital imaging and communication in medicine, and telehealth. This study focuses on several basic frameworks for chaos-based MIE. Multiple chaotic maps, robust chaos-based techniques, and fast and simple chaotic system designs of chaos-based MIE are demonstrated. The major technical notes, features and effectiveness of chaos-based MIE are investigated for future research directions. The chaotic maps of MIE are illustrated, and security evaluation methods for chaos-based MIE are explored. Design issues in the implementation of chaos-based MIE are demonstrated. The findings can inspire researchers to design an innovative, advanced chaos-based MIE system to better protect MIs against attacks and ensure robust MIE.

## 1. Introduction

Dachselt et al. [1] presented their opinion on the connection between chaos and cryptography (CRY), and many investigations have been conducted on the application of chaotic behavior to encryption. A chaotic masking (MAS) encoder consists of a chaotic encryption module, whose output chaos-based signal is added to the information signal. This sum is transmitted over a secure communication (SEC) or data encryption (DE) channel. A chaos shift keying encoder comprises two or more chaotic encryption modules with different parameters. The initial values and control parameters are used to iterate the chaotic maps to generate a real-number time series for the encryption. One of these, whose output signal sum is transmitted over the SEC or DE channel, is selected according to the discrete information signal. A chaos-based encryption algorithm is characterized by sensitive dependence on initial and chaotic system parameters, pseudorandomness, unpredictability, and a lack of periodic behavior. Jakimoski et al. [2] discussed the effects of chaotic encryption techniques on block ciphers (BCs) and presented several chaotic BCs. The highly unpredictable and random-looking trajectory of chaos-based signals is the most attractive characteristic of deterministic chaotic encryption technologies and may lead to novel CRY applications. A logistic map (LM) has been used for BCs to achieve DE and SEC. Exclusive or (XOR) operation has been integrated into LM-based BCs, and the key space (KSP) measures of these BCs have been analyzed. Medical information encryption protects information from undesirable attacks by converting it into an unrecognizable form [3]. This rapidly advancing technology can be applied to medical image (MI) storage, transmission, telemedicine, telehealth, and healthcare. Medical data encryption mainly involves scrambling (SCR) of the content of clinical biomedical data, such as text, MIs, electrocardiography (ECG), electroencephalography (EEG), peripheral capillary oxygen saturation, blood pressure, body temperature, biomedical signals, audio, and video to make clinical biomedical data unreadable, invisible, or incomprehensible during SEC. The aim is to protect clinical biomedical data against attacks. The encryption procedure may use plaintext (original biomedical signals), an encryption process, or ciphertext (encrypted biomedical signals). The characteristics of chaotic systems include sensitive dependence on initial conditions and chaotic parameters, random-like behavior, and continuous broad-band power spectral bandwidth [4]. Chaos theory has extensive applications in the field of CRY. Almost all chaos-based encryption mechanisms use dynamical and nonlinear functions that are defined on a set of real numbers, and the possible relationships between chaos and CRY have been explored. The iterations of a chaos-based map can spread from the initial region over the entire real-number time series (space), leading to the desired diffusion (DIFF) and confusion (CONF) of CRY features. Ou [5] noted the relationships between chaotic theory and CRY, and derived BCs with 128-bit symmetric keys (SKs) from an LM using appropriate parameters and initial values. Chaos-based encryption mechanisms are highly unpredictable and are suitable candidates for pseudorandom number generators (PRNGs) and massive MIs CRY.

The permutation (PER), XOR, substitution (SUB), transform (TRAN), global and local information entropy (GLIE), and neural network (NN) operations are commonly integrated into chaos-based image encryption (IE). Chaotic SKs, asymmetric keys (ASKs), BCs, and stream ciphers (SCs) have been discussed for IE [6]. A chaotic system is represented by a pseudorandom real-number time series, and unpredictable complex behaviors are exhibited in deterministic dynamical and nonlinear algorithms owing to the sensitivity to the initial values and control parameters. The integration of chaotic CRY with LM, tent map (TM), Arnold cat map (ACM), piecewise linear map (PLM), and Henon map (HM) PNG has been demonstrated to generate cipher images using XOR, PER, TRAN, and NN operations. The security evaluation methods (SECEVAMs) for IE CRY include KSP, key sensitivity (KSS), histogram analysis (HA), pixel correlation (PC), GLIE, unified averaged changed intensity (UACI), and number of pixel change rate (NPCR). IE algorithms with high encryption and decryption efficiency are required because image signals are at serious risk of privacy leakage [7]. When the amount of image information is very large, meeting the real-time (RT) encryption requirements becomes difficult. Therefore, chaotic algorithms offer significant advantages in terms of IE. The cosine map (CM), LM, TM, cosine-sine map (CSM), and logistic sinusoidal map (LSM) exhibit good chaotic dynamic performance and can be applied to IE. High-encryption strength analysis methods for the related chaotic IE have been developed to meet the requirements of high encryption security. The SECEVAMs for chaotic IE algorithms include NPCR, UACI, HA, PC, adjacent correlation coefficient (ACC), GLIE, KSP, KSS, and time complexity analysis (TCA). The optimal ACC index between plain and cipher images should be close to zero, and higher GLIE and KSP indices in cipher images are better. The optimal NPCR and UACI values are 0.9961 and 0.3347, respectively. Shorter IE runtimes in time complexity analyses are better. The Chebyshev polynomial map (CPM) can provide better protection when it is adopted for public-key CRYs, that is, the advanced encryption standard (AES), data encryption standard (DES), and Rivest-Shamir-Adleman (RSA) [8]. Chaotic encryption has a better computational efficiency for RT image and video CRY. Image CRY is described as the process of converting significant information into an unrecognizable form. Chaotic IE architecture generally includes the CONF and DIFF phases. The TM, LM, LSM, and CPM have been used for IE. Security analysis is performed to quantify the performance of encryption technologies. Security analysis consists of the differential analysis of NPCR and UACI parameters, statistical analysis of HA and PC parameters, key analysis of KSP and KSS parameters, and analysis of GLIE parameters.

The problems and effectiveness of advanced sixth-generation mobile communication schemes, which are integrated into applications and the Internet of Medical Things (IoMT), for detecting, and mitigating the spread of COVID-19 and designing healthcare infrastructures for future pandemics have been introduced [9]. Lin [10] studied several mobile telemedicine technologies. Such technologies are expected to become ubiquitous for the delivery of biomedical signals in medicine. Time-frequency feature extractions of biomedical signals, such as EEG, ECG, and speech signals using Hilbert–Huang transformation-based analysis, have also been discussed [11].

Lin et al. [12] proposed a dimensional (1D) chaotic CRY for clinical EEG signals. The 1D chaotic LM-based SCR, discard, and PER technologies were used to achieve clinical EEG visual encryption. The security level of the chaos-based encryption mechanism was evaluated using visual analysis (VA), normalized correlation coefficient (NCC), and percent root-mean-square difference (PRD) values. A two-dimensional (2D) chaotic EEG CRY has also been developed and can be applied to mobile SEC [13]. 2D chaotic LM-based SCR, discard, and PER operations have been used. The cryptanalysis schemes included VA, NCC, PRD, and bit error rate (BER). The chaotic EEG system of CRY was implemented using Microsoft Visual Studio Development Kit and C# programming language, as it can run on computers with the Microsoft Windows operating system [14]. The operations included LM, discard, SCR, XOR, and PER. The PRD and TCA parameters of the original and cipher EEG signals were explored for differential attack evaluation. Lin [15] proposed a chaos-based visual CRY method using an empirical mode decomposition (EMD) scheme for 1D clinical EEG signals. He used 2D LM-based encryption SCR, and a 2D block interleaver method to provide a robust and unpredictable intrinsic mode function (IMF)-based visual encryption algorithm. The security level of the chaos-based visual CRY mechanism was evaluated using VA, mean square error (MSE), and Pearson’s correlation coefficient (PCC) values. Table 1 lists the technical notes of the chaos-based encryption concepts.

There are many literature reviews and concept articles demonstrating chaotic text, voice, data, image, and video signal encryption. However, reviews and concept articles on chaotic medical image encryption (MIE) are rare. Therefore, this study focuses on chaotic MIEs. Medical clinical images have a larger number of bits and share similarities with video media encryption. Chaos-based encryption algorithms are characterized by their sensitive dependence on initial and chaotic system parameters, pseudorandomness, unpredictability, and lack of periodic behavior, and are suitable for the encryption of a larger number of bits. Chaos-based encryption mechanisms are highly unpredictable and are suitable candidates for pseudorandom number generators (PRNGs) and massive MIs. RT and robust chaos-based medical image encryption (MIE) systems with a high security level, large key space, high unpredictability, better bifurcation behavior, low computational complexity, and good encryption outcomes could be achieved. Chaotic MIE has become an important research area in advanced MIE strategies. Chaotic MIE technology can be used in MI storage systems, cloud-based medical systems, healthcare systems, telemedicine, mHealth, picture archiving and communication systems, digital imaging and communication in medicine, and telehealth. By revealing the basic principles of chaos-based MIE, this study aims to inspire innovative design methods in this field to improve encryption speed, accuracy, and security robustness.

This part of this article has been published by the International Conference on Advanced Communications Technology (ICACT) 2025 [16]. The remainder of this study is organized as follows. Section 2 presents chaos-based MIE methods, demonstrating the technical notes on the design issues of MIE technologies resistance to various attacks. In Section 3, discussions of the technical features and effectiveness, basic chaotic maps, and security evaluation analyses that are used for chaos-based MIE using region of interest (ROI), deoxyribonucleic acid (DNA), and artificial intelligence (AI) schemes are outlined. In Section 4, illustrations of chaos-based MIE applied to medical cloud, IoMT, mHealth, healthcare, and telemedicine are demonstrated. Finally, Section 5 and Section 6 present the discussions, and conclusions of this study.

## 2. The Study of Chaos-Based MIE

Table 2 shows the technical notes of chaos-based MIE concepts. Novel encryption/decryption technologies with perturbation (PERT), PER, and SUB operations have been proposed to protect MIs [17]. Two novel 2D chaotic map-based cipher approaches have been proposed to enhance the robustness and reduce the security breach risks for MIE. Statistical and security analysis of the MIE was performed using the NPCR (99.814%), UACI (33.694%), peak signal-to-noise ratio (PSNR) (7.723 dB), and GLIE (7.998). The mean square error (MSE), NCC, and TCA were also utilized. An improved chaotic cryptosystem with 2D LM, baker map (BM), and HM has also been developed for the rapid protection and encryption of MIs [18]. The complex chaotic PNG, PER, XOR, SCR, and SUB operations were integrated to generate a high encryption mechanism that exhibits high random behavior, high complexity, a large KSP, unpredictability, high entropy, and high-level security. SECEVAMs were performed using PSNR, HA, NCC, GLIE, KSP, KSS, NPCR, and UACI. A chaotic CRY for digital imaging and communication in medicine (DICOM) images of 16-bits has been proposed using a new enhanced chaos-based economic map (ECEM) [19]. The ECEM was designed according to an economic map, and it exhibited better bifurcation behavior, a large KSP, a better SCR operation, and positive Lyapunov exponent values. The pixel operations of PER, DIFF, SUB, MAS, SCR, and swapping (SWA) were considered to resist brute-force attacks to overcome the challenges of DICOM MIE. SECEVAMs using HA, KSS, KSP, NPCR, UACI, GLIE, and PC were analyzed. HA presented a graph of the pixel distribution of plain DICOM images, which had a meaningful regular distribution, and the HA of cipher DICOM images had a uniform distribution. The PC values of two adjacent pixels in an original image provided significant information. The PC values of the original image were high, and the original image was informative. In contrast, the PC values of the cipher image were low.

MIE technology based on a hyperbolic sine chaotic map (HSCM) with enhanced nonlinearity has been proposed [20], in which the XOR, PNG, PER, SCR, and DIFF operations were used to provide a higher security level. The performance analysis of security using the KSP, KSS, variance of histograms (VARH), NCC, NPCR, and UACI parameters was outlined in detail. The protection of patient privacy and clinical MIs has attracted considerable interest [21]. A large volume of MIs must be encrypted in RT. An MIE framework based on the BM and HM was proposed. The encryption framework included the PNG, DIFF, CONF, S-box, MAS, SUB, and XOR operations. A detailed SECEVAM was performed, including statistical analyses of the HA, PC, differential analyses of the UACI and NPCR, KSS and KSP analyses, and TCA. The MIE scheme achieved a higher security level and faster encryption speed. Multilevel chaos-based encryption with a SUB mechanism using three-dimensional (3D)-chaotic maps (SM, LM, and cubic maps (CUMs) have evolved as frameworks for enhancing the KSP, pixel DIFF, encryption speed, reliability, security, and robustness of MIs [22]. The PER (CONF) encryption methodology has also been implemented using an ACM in MIE. The CONF-DIFF and PER-SUB architectures are commonly used for MIE. CONF is widely used in CRY and is defined as the property whereby the relationship between the encryption key and cipher MI is hidden. This indicates that even with a small change in the encryption KSS, there should be a large change in the encrypted MIs. DIFF is defined as a property whereby the relationship between a plain image and a cipher image is hidden. This indicates that a small change in the plain image significantly alters the cipher image. The BC, SC, SK, and ASK mechanisms have been demonstrated in MIE. Chaos-based MIE has received significant attention for achieving RT MIE. KSS, statistical, and performance analyses have been performed using the HA, GLIE, PC, KSS, KSP, PSNR, mean absolute error (MAE), MSE, root mean square error (RMSE), and universal image quality index (UIQI) parameters and chaos-based encryption schemes have a large KSP and KSS to resist brute-force attacks.

A chaotic encryption algorithm based on the high-speed PER process of the Secure Hash Algorithm (SHA)-256 hash function (HF), and the adaptive DIFF of the Chen-based hyperchaotic map (CHCM) has been developed to protect MIs against attacks [23]. Security criteria were observed using HA, HA with the chi-square test (CSQT), PC, GLIE, KSS, KSP, PSNR, NPCR, and UACI. The chaos-based MIE mechanism exhibited excellent performance in terms of efficiency and reliability. A new MIE scheme has been presented, in which the linear feedback shift register (LFSR) was adopted as a PNG [24]. The MIs were randomized using the logistic-tent map (LTM), and SCR using the ACM. A cipher MI can be generated using the XOR, PNG, randomized, and SCR operations. MIE commonly applies SCR to remove the correlation between neighboring pixels of MIs by shifting their positions. The ACM can be easily integrated and exhibits superior ergodic properties. Encryption and decryption performances have been estimated using TCA, VARH, PC, GLIE, NPCR, UACI. KSS, PSNR, and structural similarity index measurement (SSIM). Encryption architectures using PER and DIFF have also been introduced [25]. The SK-based Latin square was used to randomize the pixels of the original image into different rows and columns, effectively decreasing the high correlations between adjacent pixels, and various images exhibited different PER effects. The four-dimensional memristive chaotic sequence (4D MCS) used in the PER and DIFF processes, and the SHA-256 HF were integrated into the MIE system. The security level of the proposed chaos-based encryption methods has been evaluated using HA, KSP, KSS, VARH, PC, GLIE, NPCR, and UACI. Chaos-based MIE has been demonstrated as highly secure and robust. An MIE scheme based on a chaotic PER-DIFF structure has been introduced [26], and LSM and LTM were adopted to enhance the KSS and generation to improve the security of MIs. A zigzag TRAN scanning operation was used to increase the robustness and efficacy of encryption technology. The DES, AES, and RSA encryption algorithms are not suitable for MIs because of the larger number of bits of MIs, low security, and low efficiency. Chaotic maps have excellent characteristics, such as randomness, ergodicity, sensitivity to the initial parameters, and chaotic control parameters. Thus, chaos-based encryption is more suitable for medical applications. The KSS, KSP, HA, CSQT, PC, ACC, MSE, NPCR, UACI, and MAE evaluation schemes have been used to confirm satisfactory security level requirements for MIE.

Securing MIs and electronic patient reports (EPRs) through chaotic encryption schemes has become a primary concern in both research and commercial fields [27]. CRY of DICOM images and EPRs has been performed using the LM, XOR, and nonlinear feedback shift register (NLFSR) operations. The HA, SSIM, PSNR, NCC, KSP, KSS, GLIE, RMSE, NPCR, and UACI analysis methodologies have been used to validate the chaotic cipher. Advanced lightweight MIE technology includes the LM, Brownian motion, XOR, PNG, and CHCM operations [28]. MIE schemes can be implemented using the PER (CONF) and SUB (DIFF) approaches. The encryption performance of MIE technologies has been investigated using HA, ACC, NPCR, UACI, MSE, PSNR, GLIE, and TCA. The histogram of the cipher MI should have a uniform distribution and should not have peak values. An innovative and rapid MIE technology using chaotic LM and PNG has been developed [29]. The PER and SUB of the pixels in the MI were calculated using chaotic LM and XOR operations. Cryptanalysis of the proposed algorithm, in which the KSP, TCA, HA, MSE, PSNR, NCC, KSS, NPCR, UACI, and GLIE were used, was performed to verify the effectiveness and robustness of the technology. The results indicated that the MIE technology exhibits excellent security. An efficient and encrypted MIE method in which the SUB, DNA, LM, PNG, TM, CONF, and XOR operations are adopted has been reported [30]. Many cryptanalysis methods based on chaos theory have been used, including the KSP, KSS, HA, NPCR, UACI, PC, NCC, GLIE, and PSNR.

SC-based chaotic LM and CPM encryption schemes with SCR technology have been shown to achieve a higher encryption level in MIE and possess a better anti-attack ability [31]. An analysis using the MIE visual effect, KSS, and NPCR demonstrated that MI information could be protected more effectively. The RT and robust CRY methodology of MIE was employed across two steps of CON by rearranging array indices adopting a TM and establishing an S-box algorithm generated from the 2D HM [32]. The Lyapunov exponents and bifurcation diagrams of the chaotic dynamic behaviors for the S-box were demonstrated. The pseudorandom bit generator (PRBG) was constructed based on the DIFF approach. The SCR, XOR, and PER operations were utilized in the MIE. The SECEVAMs focused on information entropy (InE), HA, ACC, UACI, NPCR, KSP, KSS, and TCA. The MIE satisfied good encryption efficiency and exhibited resilience against differential and statistical attacks. Lightweight MIE requires a more secure and efficient encryption algorithm [33]. A plaintext-related and ciphertext feedback mechanism (PRCFM) has been innovated using a 1D sine arcsin chaotic map (SACM) for the SCR-DIFF CRY approach to resist plaintext attack. The 1D SAM chaotic map had a larger chaotic range and enhanced dynamic complexity. PER and SHA-256 operations were integrated into the encryption framework. The SECEVAMs involved KSP, MSE, PSNR, HA, ACC, InE, NPCR, UACI, KSS, and TCA. The CRY system provided acceptable performance in both encryption time and robust security. The Tent-May chaotic map (TMCM) was designed to generate PRBG as BC encryption [34]. The S-box and XOR operations were combined into the BC encryption, enhancing security and increasing the strengths of TMCM. The BC encryption involved block-wise SUB and PER. Security performance analysis included homogeneity analysis (HOA), energy, contrast, ACC, InE, MSE, RMSE, PSNR, average difference (AD), maximum difference (MD), structural content (STC), NCC, normalized absolute error (NAE), NPCR, UACI, and blocked average changing intensity (BACI). The MIE was able to combat statistical attacks and had better accuracy and security.

A new five-dimensional (5D) multi-band multi-wing chaotic map (MBMWCM) has been adopted in the MIE system, and a 5D MBMWCM with a relatively large Lyapunov exponent has been used to generate chaotic encryption sequences [35]. Quick response (QR) decomposition, XOR, and bit-level SCR operations were integrated into the MIE system, and InE, KSP, HA, KSS, NPCR, UACI, and ACC of the security parameters were evaluated.

The KSP of the MIE was relatively large, the KSS was relatively strong, and it resisted statistical attacks better for medical images. The chaos-based JPEG2000 MIE has been developed, with JPEG2000 being a wavelet TRAN-based MI format [36]. 2D chaotic LM phase SCR, chaotic PER, and single-layer chaos-based MI pixel SCAN operations were performed. The security evaluation methods of VA, NCC, and PSNR were adopted.

## 3. Chaos-Based MIE Using ROI, DNA, and AI Schemes

Table 3 shows the technical notes of chaos-based MIE using the ROI, DNA, and AI schemes. Traditional chaos-based maps with problems relating to small chaotic ranges suffer from blank and stable windows [37]. An advanced MIE algorithm using an improved cosine fractional chaotic map (ICFCM) has been proposed, which has a wider and more uniform chaotic range, a more positive Lyapunov exponent, and non-blank and non-stable windows. This technology consists of four steps for encrypting an image. In the first step, three intermediate keys are produced. In the second step, three chaotic sequences are generated using the novel ICFCM, tangent over cosine map (ToCCM), and CPM. The third step integrates DNA encoding and DIFF using the DNA operation. Finally, in the fourth step, the three modules of the color cipher image are generated using the DNA addition operation between the DNA encoded image color modules, and distinct DNA encoded chaotic sequences for each module. DNA operations can improve the security of CRY algorithms. The features of DNA operations include huge storage, low power requirements, and extreme parallelism, and they can be integrated into chaos theory. The PC, ACC, KSP, bit correct ratio (BCR), MSE, PSNR, TCA, GLIE, NPCR, and UACI were used as the cryptanalysis parameters in the above study.

MIs can be divided into the ROI and the region of background (ROB) [38]. The ROI is the part of an MI with diagnostic information, and the ROB is the part without visual or medical significance. Full and semi-full MIE modes using a variable-dimension chaotic map-based LM have been proposed. The 2D chaotic modified LM (MLM), SHA-256 HF, ROI, ROB, PER, and SUB operations were integrated into the semi-full CRY mode. The PER operation was used to encrypt the ROI, and the SUB operation was used to encrypt the entire image. The 3D/4D MLM, SHA-256 HF, PER, and SUB operations were integrated into the full CRY mode. CONF, which is used to change the pixel positions in medical images, has also been denoted as “PER”, “shuffle”, or “SCR”, in different articles, whereas DIFF, which is used to change the value of pixels in MIs, has also been defined as “SUB” or “MAS”. The GLIE, NCC, KSS, KSP, NPCR, UACI, and TCA parameters were analyzed to evaluate the effectiveness of the cipher image. The analysis results showed that the semi-full CRY mode exhibited good security performance and a lower time complexity, whereas the full CRY mode exhibited better security performance and a higher time complexity. Zhang et al. [39] proposed an MIE method that focuses on the ROI within massive MIs. The ROI and ROB were segmented from the MIs. The ROIs of the massive MIs were encrypted using Lorenz hyperchaotic maps (LHM) and the HM, XOR, SCR and DIFF operations. HA, VARH, GLIE, PC, ACC, NPCR, UACI, SSIM, KSP, KSS, and TCA were employed for the evaluation of the massive MIs. The chaos-based massive MIE technology used 13 parameters in the encryption approach, and the calculation accuracy of the 64-bit double precision data was $10^{-15}$. Therefore, the KSP value of the massive MIE was ${\left(10^{15}\right)}^{13}=10^{195},$ thereby exhibiting higher security and encryption efficiency.

Chaotic CRY systems exhibit the encryption characteristics of unpredictability, pseudorandomness, and initial value sensitivity [40]. 1D chaotic maps, such as the LM and sine map (SM), have small parameter ranges and limited chaotic behavior. Multidimensional chaotic maps have long generation times and are expensive. A bifurcation diagram and the Lyapunov exponent of the LSM showed that it has good CRY characteristics. The LSM has a shorter operation time complexity, a larger encryption parameter range, better randomness and better KSP. The clinical image had a high correlation between the image pixels and the centralized distribution of the clinical image information. The intra- and inter-block SCR schemes were adopted to encrypt the ROI and the entire image, respectively. The chaotic time sequences performed by the LSM were utilized for each pixel value after the nonlinear operation to achieve the SUB effect. The hash operation of the SHA-512 HF was integrated into the clinical IE system. The encryption effects related to the KSP, KSS, HA, PC, ACC, GLIE, NPCR, UACI, histogram uniformity (HU), and TCA parameters were compared. The analysis results demonstrated that the LSM-based MIE system has high encryption efficiency, can achieve good practicality, and is resistant to attack. MIE should be as efficient as possible [41]. Low computational complexity technologies have been proposed to secure MIs, from which the BC-based ROI and ROB operations have been extracted. The significant ROI was encrypted using the PER operation with the ACM and angle value. Subsequently, the duffing map (DM) and XOR operations were used to achieve the DIFF function for the PER-based ROI, and the ROB was not encrypted. A performance evaluation of the chaotic MIE system was performed using the HA, GLIE, MSE, NPCR, UACI, SSIM, BER, NCC, PSNR, KSP, and TCA. Demirkol et al. [42] integrated a new nonlinear memristor-based chaotic circuit (MCC), DNA coding scheme, XOR operation, and DIFF-CONF method to design an RT MIE system applied to the healthcare industry. Sufficient security was provided for the SEC. The randomness of the new nonlinear MCC-based chaotic time sequences was verified by their chaos-based dynamic behavior using the demonstration of the phase portraits, Lyapunov exponents, and the bifurcation diagrams. The main advantages related to the use of the DNA coding scheme were the large KSP, and low computational power. The evaluation metrics included TCA, HA, UACI, NPCR, and GLIE. It was suitable for MIE purposes, with resistance against various attacks, and high plaintext sensitivity was achieved.

Most MIE algorithms have demonstrated the encryption MIs in common formats; however, specific and MI formats are often not considered [43]. DICOM MIs have a large volume and high computational complexity, and efficient and secure CRY methods are an important challenge for DICOM MIs. The 3D spatiotemporal coupled chaotic lattice map (SCCLM) has been adopted for DICOM MIE, providing the complexity of chaotic sequences and enhancing the robust function of MIE. Double-layer random DNA encoding DIFF process, fractal random PER process, and SHA-256 operation have been combined into the CRY system. Security performance evaluations of HA, MSE, PSNR, SSIM, ACC, InE, KSP, NPCR, UACI, and KSS have been discussed. The CRY algorithm has been shown to perform better in solving plaintext, statistical, and differential attacks, and it can be used in the field of MIE.

A selective region MIE approach based on the cosine-cubic cascaded map (CCCM) has been proposed [44]. The CRY system consists of two stages: ROI CRY and full MIE. CCCM had better KSP, and fast computation and low complexity of low-dimensional chaotic characteristics were achieved. Edge detection was utilized on MIs to generate the ROI. The modified 2D Joseph traversal CONF mechanism was combined to SCR the positions of pixels in each sub-block, and further changed their pixel values with a round of DIFF. The block V-shape SCR scheme was used to SCR the entire image. SHA-512 HF was integrated into the CCCM-based MI CRY system. KSP, KSS, InE, local InE, HA, ACC, NPCR, and UACI were performed. An ROI-based selective lightweight MI cipher has been proposed using PLM [45], limiting hackers’ practical use of smart devices. The characteristics of smart devices include limited memory, low power, and low computing capability. PER-DIFF, XOR, bit-level circular shifts, and pixel shuffling operations were integrated into the encryption approaches. The ACC, GLIE, KSP, NPCR, UACI, TCA, and PSNR related to security and protection were calculated. Multiple MIs of arbitrary quantities and sizes can be encrypted using the enhanced security chaos-based tent-logistic cross mixed coupled map lattice (TLCMCML) [46]. The innovative TLCMCML has better chaotic features, high randomness, high unpredictability, ergodicity, and a larger chaotic range. The new MIE algorithm includes two approaches. The first approach, the odd-even interleaving PER scheme, is adopted to individually scramble the ROI in each MI; in the second approach, a synchronous scrambling, SHA-512, bit-level PER-DIFF method is used. Therefore, simultaneous SCR and DIFF operations are applied to each image. KSP, KSS, GLIE, ACC, NPCR, UACI, and TCA metrics have been evaluated, and security analysis showed that the innovative MIE algorithm has excellent CRY effectiveness and provides high-security MIE technologies.

Takagi–Sugeno–Kang Fuzzy Wavelet Brain Cereberal Controller (TSKFWBCC)-based decryption approaches have been demonstrated for MIs [47]. 4D hyperchaotic map (HCM), and XOR operation were integrated into a highly robust and precise secure MIE algorithm. The proposed encryption method exhibited excellent HA, GLIE, and PSNR performance metrics. With the rapid advancement of artificial intelligence (AI) schemes, chaos-based AI MIE has become a significant research topic in MI privacy protection [48]. High-dimensional features have been adaptively extracted from the original MIs using ResNet, and more flexible and secure CRY models have been achieved. The output of the ResNet model is encrypted using chaotic LM and XOR-SCR operations. Security evaluation methods involve ACC, InE, and HA, and the chaotic ResNet-LM MIE system can achieve higher security and RT encryption.

## 4. Chaos-Based MIE Applied to Medical Cloud, IoMT, mHealth, Healthcare, and Telemedicine

Table 4 shows the technical notes of chaos-based MIE applied to the medical cloud, IoMT, mHealth, healthcare, and telemedicine. MIs are widely used in modern clinical diagnosis and treatment. MIs containing confidential information relating to patients’ privacy are stored and transmitted using cloud-based networks [49]. SCR is performed on the plain image, and a cipher image is generated. Chen et al. [49] developed an adaptive MIE algorithm based on LSM chaotic mapping to increase the robustness of existing chaotic MIE technology. SECEVAMs using the HA, KSP, KSS, GLIE, NPCR, UACI, and NCC were employed, and the chaos-based MIE schemes were able to effectively protect against attacks, with better security and robustness. The PNG and linear congruential generator (LCG) were used with a 2D logistic coupled chaotic map (LCCM) to protect the patient confidentiality of DICOM images during storage and transmission on a cloud-based platform [50]. The XOR, CONF, DIFF, pixel MAS, and SCR encryption operations were performed, and the KSS, HA, PC, GLIE, NPCR, and UACI SECEVAMs were presented in detail. A synchronization chaotic approach with a fast-reaching finite time has also been designed in the transceiver, as an encrypted key generator for a fourth-order Runge–Kutta chaotic system (FORKCS) [51]. An XOR operation was performed to protect patients’ MIs. HA, VARH, PC, GLIE, NPCR, UACI, and TCA were applied to analyze the security and robustness of the chaos-based MIE.

The storage and transmission of digital MIs for detecting and treating diseases increased significantly in hospitals during the COVID-19 pandemic [52]. A 1D LM is a basic method that is easy to implement. MLMs were developed to increase unpredictability and encryption robustness. This novel encryption approach comprises three main steps. In the first step, a variable-length gray-level code, and an XOR operation are integrated into the generation of the encrypted key to CONF the intruder and subsequently used as the initial parameters of both modified LM. In the second step, the one-stage image pixels undergo PER using the address code that is generated from the sorting TRAN of the first MLM. Finally, a complete DIFF procedure is applied to the entire image using the second MLM to overcome differential and statistical attacks. The encryption efficiency was determined using the PC, ACC, NPCR, UACI, KSS, and KSP parameters. The analysis results indicated that the chaos-based MIE exhibits high encryption performance and robust resistance to various attacks. One of the aims of digital innovation is the design and implementation of MIE with a highly efficient and resilient SEC for smart telehealth [53]. Framework-based Hessenberg TRAN and chaotic encryption have been applied to protect security and decrease computational time while encrypting MIs. The IEFHAC algorithm comprises three operations: XOR, rotated (ROT), and PRBGs. The LM and SM were integrated into IEFHAC to achieve efficient CONF and the Hessenberg household transform was used to achieve DIFF efficiency. The chaotic LM and SM sequences made the MIE system strong in combating attacks. The IEFHAC scheme was evaluated in terms of HA using the CSOT, NPCR, UACI, KSP, KSS, SSIM, NCC, BER, and TCA. The analysis was performed on an Intel, Core i3-3120M, CPU @ 2.5 GHz with 4 GB of RAM using Windows 10. The security analysis demonstrated that the IEFHAC algorithm provides computationally efficient solutions for MIE.

The SEC of MIs is an important requirement in telemedicine and cloud-based healthcare systems [54]. Adaptive frameworks for protecting the encryption and confidentiality of MIs are transmitted through the cloud-based medicine system. Three-dimensional chaotic PLMs are adopted to generate a PNG keystream, which is used to perform 8-bit (pixel) and 2-bit DNA and PERs operations on MIs. XOR and DIFF operations are integrated into the chaos-based MIE to enhance the security. The HA, VARH, NCC, PSNR, NPCR, UACI, and TCA are used to evaluate the encryption performance. Medical clinical images play an important role in disease diagnosis, and protecting medical clinical images is a significant design challenge in mHealth, e-healthcare and telemedicine with RT application [55]. The novel dynamic circular bit shifting and bit-flipping methods in the CONF-DIFF phase of MIs provide better security and confidentiality. Three-dimensional chaos-based hyperbolic maps (HYM) are utilized to generate three chaotic sequences with XOR operation for MIE, and the encryption has the characteristics of robustness, higher randomness, high efficiency, higher complexity, unpredictable behavior, larger KSP, and KSS. SECEVAMs include the GLIE, ACC, MSE, NPCR, UACI, SSIM, feature-similarity (FSIM), NAE, PSNR, MD, and STC. The massive MIE plays a crucial role in the secure sharing, storing, and transmission of MIs [56]. ROI and non-ROI of MI design aspects have been integrated into the proposed MIE algorithm. The random pixel indices of the non-ROI of MI were encrypted using a 1D TM. A 3D LHM was utilized in the CONF encryption process of ROI-MI with the widely used KSP. Assuming the encryption key parameters are 14-digit precision, the A 3D LHM has six encryption key parameters; therefore, the A 3D LHM had the widely used KSP of $10^{14\times 6}$. The DNA-encoding DIFF process and XOR operation were used in the ROI-MI encryption mechanism. Security performances were measured using KSP, HA, ACC, InE, MSE, PSNR, signal-to-noise ratio (SNR), maximum deviation (MDE), irregular deviation (ID), and NPCR. The robust MIE framework can be applied to the fields of EPR, DICOM, cloud-based mHealth, and telemedicine systems. Binary COVIDOA-based chaotic CM and LM have been utilized to generate optimal initial sequences related to the CONF and DIFF approaches of MIE [57]. The MIE algorithm had high security and computational efficiency and could be used in IoMT-based healthcare systems. Security analyses of HA, InE, ACC, UACI, NPCR, MSE, and PSNR were performed.

In the era of mHealth and telemedicine, X-ray MIs have been encrypted using public-key CRY [58]. Elliptic curve CRY (ECC) and Blum-Goldwasser CRY (BGC) were combined with the CRY mechanism to achieve superior encryption time performance. ECC had the mathematical features of elliptic curves, and it provided extensive CRY protection on small key lengths and fast encryption transactions. The BCG encryption methodology was designed through Blum Blum Shub (BBS) PRBG for the XOR-based MI stream cipher. The characteristics of the ECC and BCC were unpredictability and fortifying CRY issues. Pixel randomization of X-ary MIs was adopted through a JoanS-MuraliP’s chaotic map (JSMPCM), which was developed by combining the LM and a quadratic map (QM). The JSMP chaotic map had a large KSP. The security metric performance of the InE, ACC, NPCR, UACI, and TCA was demonstrated. MI exhibited sensitive patient information, and reliable and efficient MIE is required for telemedicine [59]. The simultaneous PER and DIFF framework (SPDF) was developed using a 1D Chebyshev iterative chaotic map (CICM) with infinite collapse, and the modified Josephus traversing method. The CPM and CICM have been integrated into the 1D CICM with infinite collapse, resulting in higher unpredictability and ergodicity. The dynamic SCR scheme was adopted into the modified Josephus traversing method, enhancing PER operation. SECEVAMs of KSP, HA, ACC, NPCR, UACI, InE, PSNR, SSIM, and TCA were conducted. The MIE had high efficiency and could withstand the chosen plaintext and noise attacks. A dynamic ROT MIE based on an improved Lorenz chaotic map (ILOCM) is innovative for solving the security problems for the digital MIs in IoMT transmission [60]. The ILOCM included new nonlinear adjustment terms and encryption parameters. The ILOCM had a wider range of chaotic parameters and better chaotic characteristics, and the ILOCM-based PRBG was more unpredictable. The ILOCM-based key generator applied the MIE scheme to achieve dynamic rotation PER and double SCR-DIFF. KSP, MSE, InE, HA, ACC, PSNR, SSIM, NPCR, UACI, and TCA were discussed. A new dynamic block cipher of MIE technology using a time-delay chaotic map (TDCM) was developed for the PACS in the hospital [61]. XOR operations were adopted for the BC, and ACCs were analyzed to illustrate the effectiveness and feasibility of the TDCM-based cipher. Chaos-based symmetric MI ciphers were designed using a tent logistic tent map (TLTM) and HM for an e-healthcare platform [62], and PER and DIFF operations were adopted to pixel SCR. Security features of KSP, KSS, ACC, HA, GLIE, NPCR, UACI, PSNR, MSE, and TCA were obtained. The analysis results showed that the TLTM-based MIE was efficient and robust. An MI watermarking and encryption security method has been revealed for smart healthcare systems [63]. 4D HCM, 2D Henon–sine map (HSM), SCR, PER–DIFF, and XOR operations were used for the MI stream cipher. Security analysis of the proposed methods was verified using NPCR, UACI, PSNR, MSE, GLIE, and TCA. A 1D chaotic cosine–sine map (CSM) has been developed with better complex behavior, better randomness in nature and a larger chaotic range [64], and it can be used in MIE systems in healthcare. MIs such as X-rays, magnetic resonance imaging (MRI) scans, computed tomography (CT) scans and ultrasound images (UIs) were protected by integrating PERT, DIFF, and XOR operations. Security analysis included NPCR, UACI, ACC, GLIE, KSS, BCR, MSE, and PSNR values.

Chaos-based encryption methods were used to maintain the security of MIs and ensure that only the AU user could access sensitive MIs. DICOM MIs have been widely used in the fields of medical diagnostics process, telemedicine, and healthcare, and online DICOM MIE methods are required [65]. Effective DICOM MIE approaches using XOR operations with a secret process derived from an LHM were implemented, and high-security multi DICOM MI access was verified. Security analysis included MSE, SNR, PSNR, ACC, and Q value. Block chain–based chaotic deep generative adversarial network (BCDGE) encryption technology has been constructed to achieve advanced levels of security overcoming hackers for IoMT-based MIs [66].

A blockchain scheme was adopted to enhance MI security, and a chaotic deep generative adversarial network-based CRY technology was applied to generate a private key with a larger KSP, excellent randomness, complexity, and high entropy. SHA-1 hash algorithm, XOR, and SCR–DIFF operations were utilized in the chaotic MIE system. Security analysis included KSP, InE, HA, UACI, NPCR, SSIM, MSE, ACC, and TCA parameters. The primary concern related to MI is the privacy and security approaches with cloud-based transactions and safe web [67]. The distributed and immutable feature of block chain infrastructure was provided using the chaotic TM MI cipher–resistant brute–force attacks.

Three encryption key parameters were adopted in the chaotic TM; therefore, it had a larger KSP and KSS, high unpredictability, and high complexity. Chaotic SM, XOR, PER, SHA-256, and scattered operations were integrated into the chaos-based MIE. Security analysis of HA, SIE, NPCR, UACI, MSE, PSNR, KSP, KSS, ciphertext difference rate (CDR), and complexity of computation (CC) were explored. A block chain scheme has been adopted to decentralize the storage and transmission of encrypted MIs through the network [68]. The block chain framework created accountability and control across all independent smart devices, and chaos-based MIE was utilized in secured management in the IoMT-edge system. MIs were decomposed into R, G, and B, in which these sub-images were SCR using multi-scroll maps (MSMs), HM, and ACM, respectively. The MSMs had more intricate dynamic behavior and generated highly unpredictable chaos-based time sequences. The evaluation metrics of security strength included NPCR, UACI, ACC, InE, and TCA.

## 5. Discussion

MIs include CT scans, MRI scans, positron emission tomography (PET) scans, UIs, X-rays, and so on. Table 5 displays the important technical features and effectiveness of chaos-based MIE. There are many technical features and different levels of effectiveness concerning the operations commonly used in chaos-based MIE, such as AI, fuzzy, NN, ASK, AU, BC, block chain, cloud, CONF, CT, DICOM, DIFF, discard, DNA, EPR, healthcare, HF, IoMT, LCG, lightweight, LFSR, NLFSR, MAS, MRI, mobile, PACS, PER, PET, PERT, PNG, PRBG, ROB, ROI, ROT, RT, Sbox, SC, SCR, SEC, SK, storage, telemedicine, telehealth, transmission, SUB, SWA, TRAN, ultrasound, X-rays, XOR, and so on. The list of abbreviations is shown on the pages 25–29. Table 6 shows the important chaotic maps of MIE. These chaotic maps are used in chaos-based MIE, such as 2D HM, 2D HSM, 2D LM, 2D LCCM, 2D MLM, 3D CM, 3D HYM, 3D LHM, 3D MLM, 3D PLM, 4D HCM, 4D MCS, 4D MLM, ACM, BCDGE, BM, CHCM, CICM, CM, CCCM, CPM, CSM, CUB, ECEM, FORKCS, HM, HCM, HSCM, ICFCM, ILOCM, JSMPCM, LM, LCCM, LHM, LSM, LTM, MCC, MDCM, MBMWCM, MLM, MSM, PLM, QM, SCCLM, SM, SAM, TDCM, TLCMCML, TLTM, TM, TMCM, TOCCM, and so on.

Table 7 lists the SECEVAMs for chaos-based MIE. The basic security evaluation methods include ACC, AD, BACI, BCR, BER, CC, CDR, CSQT, EDR, energy contrast, FSIM, GLIE, HA, HOA, HU, ID, InE, KSP, KSS, local InE, MAE, MD, MDE, MSE, NAE, NCC, NPCR, PC, PCC, PRD, PSNR, RMSE, STC, SIE, SSIM, SNR, STE, Q-value, TCA, UACI, UIQI, VA, VARH, and so on. In this study, we focused on chaos-based MIE, which is critical in clinical MIE operations. All of the aforementioned technologies have been significant in the advancement of chaos-based MIE. Relevant studies focusing on the following aspects were deemed significant:

- Chaos-based MIE operations and applications;
- Chaos-based maps;
- Chaos-based MIE cryptanalysis (SECEVAMs).

## 6. Conclusions

**Figure 1:**
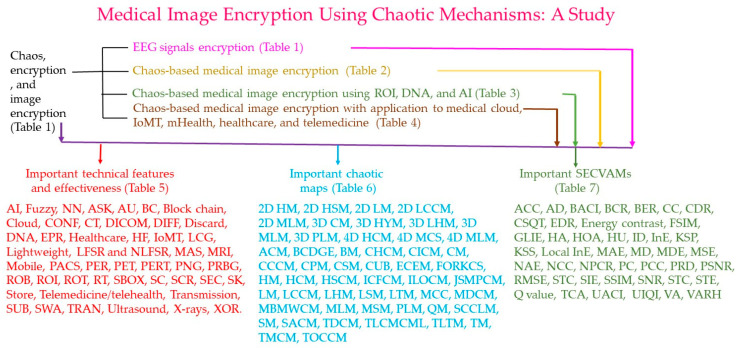
Principal categories of the studies on chaotic MIE.

MIs are stored and transmitted through networks, and strong and robust encryption algorithms are essential. The main objective of this study was to present innovative approaches for chaotic MIE. Studies on the chaotic MIEs are rare. The fundamental knowledge outlined in this study plays a significant role in the development of chaotic MIE. We examined the basic concepts of modern CRY published in research between 2001 and 2025 concerning chaos-based MIE based on the Web of Science journal papers (journal citation reports 2023). The principal categories of the chaos-based MIE studies are shown in Figure 1. The surveyed studies relating to chaos, CRY, and IE techniques included eight papers [1,2,3,4,5,6,7,8], relating to chaos-based EEG signal encryption included four papers [12,13,14,15], and relating to chaos-based MIE included twenty papers [17,18,19,20,21,22,23,24,25,26,27,28,29,30,31,32,33,34,35,36]. The original literature relating to ROI, DNA, and AI schemes included twelve papers [37,38,39,40,41,42,43,44,45,46,47,48], and the original literature relating to medical cloud, IoMT, mHealth, healthcare, and telemedicine techniques included twenty papers [49,50,51,52,53,54,55,56,57,58,59,60,61,62,63,64,65,66,67,68].

Chaotic MIE has been applied to mHealth, telehealth, telemedicine, IoMT, and cloud-based medicine systems. This is a conceptual article, guiding beginners to understand the design principles of chaotic MIE. MIE involves a larger data volume, and shares similarities with video media encryption.

The design challenges of MIE include designing fast, accurate, and robust encryption mechanisms for future applications. By revealing the basic principles of chaos-based MIE, this study aims to inspire innovative design methods in this field to improve encryption speed and security robustness. Advanced MIE methods can be implemented to overcome plaintext, statistical, and differential attacks, and can be used in the field of MIE.

## Figures and Tables

**Table 1:** Technical notes on the chaos-based encryption concepts.

References	Technical Notes
Dachselt et al. [1]	Chaos and CRY; Chaotic behavior and encryption; SEC or DE; Chaotic encryption modules with different parameters; Initial values and control parameters; Chaotic maps; Real-number time series.
Jakimoski et al. [2]	The effects of chaotic encryption techniques; Several chaotic BCs; The highly unpredictable and random-looking trajectory of chaos-based signals; Novel CRY applications; XOR operations; LM-based BCs; KSP measures of BCs.
Yang et al. [3]	Protecting information from undesirable attacks by converting it into an unrecognizable form; MI storage, transmission, telemedicine, telehealth, and healthcare; SCR of the content of clinical biomedical data; SEC; Protecting clinical biomedical data against attacks; The encryption procedure may use plaintext, an encryption process, or ciphertext.
Kocarev et al. [4]	Sensitive dependence on initial conditions and chaotic parameters, random-like behavior, and continuous broad-band power spectral bandwidth; Dynamical and nonlinear functions; A set of real numbers; The possible relationships between chaos and CRY; The desired DIFF and CONF CRY features
Ou et al. [5]	The relationships between chaotic theory and CRY; BCs with 128-bit SKs; An LM with appropriate parameters and initial values; Highly unpredictable; Suitable candidates for PNGs and CRY methods
Zhang et al. [6]	PER, XOR, SUB, TRAN, GLIE, and NN operations; Chaos-based IE; Chaotic SKs, ASKs, BCs, and SCs; A pseudorandom real-number time series, and unpredictable complex behaviors are exhibited in deterministic dynamical and nonlinear algorithms owing to the sensitivity to the initial values and control parameters; Chaotic CRY with LM, TM, ACM, PLM, and HM PNG; XOR, PER, TRAN, and NN operations; SECEVAMs: KSP, KSS, HA, PC, GLIE, UACI, and NPCR.
Fang et al. [7]	IE algorithms with high encryption and decryption efficiency; If the amount of image information is very large, meeting the RT encryption requirements becomes difficult; The CM, LM, TM, CSM, and LSM, exhibit good chaotic dynamic performance; High encryption strength analysis methods; Meeting the requirements of RT encryption and high encryption security; SECEVAMs: NPCR, UACI, HA, PC, ACC, GLIE, KS, KSS, and TCA.
Zia et al. [8]	The CPM can provide better protection when it is adopted for public-key CRYs, such as, AES, DES, and RSA; Chaotic encryption has a better computational efficiency for RT image and video CRY; Chaotic IE architecture generally includes the CONF and DIFF phases; The TM, LM, LSM, and CP have been used for IE; SECEVAMs: differential analysis of the NPCR and UACI parameters, statistical analysis of the HA and PC parameters, key analysis of the KSP and KSS parameters, and analysis of the GLIE parameters.
Lin et al. [12]	1D chaotic encryption of clinical EEG signals; SCR, discard, and PER technologies were used to achieve clinical EEG visual encryption; SECEVAMs: VA, NCC, and PRD;
Lin et al. [13]	2D chaotic EEG encryption; Mobile SEC; 2D chaotic LM-based SCR, discard, and PER operations; SECEVAMs:VA, NCC, PRD, and BER.
Lin et al. [14]	Chaotic EEG system of encryption; Microsoft Visual Studio Development Kit and C# programming language; Run on computers with Microsoft Windows operating system; LM, discard, SCR, XOR, and PER operations; SECEVAMs: PRD, and TCA.
Lin [15]	The chaos-based visual CRY method; EMD scheme; 2D clinical EEG signals; 2D LM-based encryption SCR; 2D block interleaver method; Robust and unpredictable IMF-based visual encryption algorithm; SECEVAMs: VA, MSE, and PCC.

**Table 2:** Technical notes on the chaos-based MIE concepts.

References	Technical Notes
Yasser et al. [17]	Novel encryption/decryption technologies; PERT, PER, and SUB operations; Protect MIs; Two novel 2D chaotic map-based cipher approaches; Enhance the robustness and reduce security breach risks; SECEVAMs: NPCR, UACI, PSNR, GLIE, MSE, NCC, and TCA.
Gafsi et al. [18]	An improved chaotic cryptosystem with 2D LM, BM, and HM; Rapid protection and MIE; Complex chaotic PNG, PER, XOR, SCR, and SUB operations; Generate a high encryption mechanism that exhibits high random behavior, high complexity, a large KSP, unpredictability, high entropy, and high-level security; SECEVAMs: PSNR, HA, NCC, GLIE, KSP, KSS, NPCR, and UACI.
Parvees et al. [19]	A chaotic CRY for DICOM images of 16-bits; A new ECEM; The ECEM was designed according to the economic map; It exhibited better bifurcation behavior, a large KSP, a better SCR operation, and positive Lyapunov exponent values; The pixel operations of PER, DIFF, SUB, MAS, SCR, and SWA; Resists brute-force attacks to overcome the challenges of DICOM MI; SECEVAMs: HA, KSS, KSP, NPCR, UACI, GLIE, and PC.
Liu et al. [20]	MIE technology based on aHSCM with enhanced nonlinearity; XOR, PNG, PER, SCR, and DIFF operations; Provides a higher security level; SECEVAMs: KSP, KSS, VARH, NCC, NPCR, and UACI.
Ibrahim et al. [21]	Protection of patient privacy and clinical MIs; A large volume of MIs must be encrypted in RT; An MIE framework based on the BM and HM; PNG, DIFF, CONF, S-box, MAS, SUB, and XOR operations; SECEVAMs: HA, PC, UACI, NPCR, KSS, KSP, and TCA; A higher security level and faster encryption speed.
Banday et al. [22]	Multilevel chaos-based encryption with a SUB mechanism; 3D-chaotic maps (SM, LM, and CUM); Enhancing the KSP, pixel DIFF, encryption speed, reliability, security, and robustness of MIs; The PER (CON) encryption methodology was also implemented using an ACM in MIE; CONF-DIFF and PER-SUB architectures; BC, SC, SK and ASK mechanisms; RT; SECEVAMs: HA, GLIE, PC, KSS, KSP, PSNR, MAE, MSE, RMSE, and UIQI; A large KSP and KSS to resist brute-force attacks.
Moafimadan et al. [23]	A chaotic encryption algorithm based on the high-speed PER process of the SHA 256 (HF), and the adaptive DIFF of the CHCM; Protect MIs against attacks; SECEVAMs: HA, CSQT, PC, GLIE, KSS, KSP, PSNR, NPCR, and UACI; Excellent performance in terms of efficiency and reliability.
Deb et al. [24]	The LFSR was adopted as a PNG; The MIs were randomized using the LTM, and SCR using the ACM; A cipher MI can be generated using the XOR, PNG, randomized, and SCR operations; Exhibits superior ergodic properties; SECEVAMs: TCA, VARH, PC, GLIE, NPCR, UACI. KSS, PSNR, and SSIM.
Chai et al. [25]	Encryption architectures using PER and DIFF; The 4D memristive chaotic sequences used in the PER and DIFF processes; SHA-256 HF; SECEVAMs: HA, KSP, KSS, VARH, PC, GLIE, NPCR, and UACI; Highly secure and robust.
Kumar et al. [26]	A chaotic PER-DIFF structure; LSM, and LTM were adopted to enhance the KSS and generation to improve the security of MIs; A zigzag TRAN scanning operation was used to increase the robustness and efficacy of the encryption technology; Chaotic maps have excellent characteristics, such as randomness, ergodicity, sensitivity to the initial parameters, and chaotic control parameters; SECEVAMs: KSS, KSP, HA, CSQT, PC, ACC, MSE, NPCR, UACI, and MAE; Satisfactory security level requirement for MIE.
Das et al. [27]	Securing MIs and EPRs; CRY of DICOM images and EPRs was performed using the LM, XOR, and NLFSR operations; SECEVAMs: HA, SSIM, PSNR, NCC, KSP, KSS, GLIE, RMSE, NPCR, and UACI.
Masood et al. [28]	Advanced lightweight MIE technology; LM, Brownian motion, XOR, PNG, and CHCM operations; PER (CONF) and SUB (DIFF) approaches; SECEVAMs: HA, ACC, NPCR, UACI, MSE, PSNR, GLIE, and TCA.
Kumar et al. [29]	An innovative and rapid MIE technology using chaotic LM and PNG; The PER and SUB of the pixels in the MI were calculated using chaotic LM and XOR operations; SECEVAMs: KSP, TCA, HA, MSE, PSNR, NCC, KSS, NPCR, UACI, and GLIE; The MIE technology exhibits excellent security.
Barik et al. [30]	An efficient and encrypted MIE method; SuB, DNA, LM, PNG, TM, CONF, and XOR operations; SECEVAMs: KSP, KSS, HA, NPCR, UACI, PC, NCC, GLIE, and PSNR.
Liu et al. [31]	SC-based chaotic LM and CPM encryption schemes; SCR technology achieved a higher encryption level; Better anti-attack ability; SECEVAMs: VA, KSS, and NPCR; The MI information could be protected more effectively.
Podder et al. [32]	The RT and robust CRY; Two steps of CON by rearranging array indexes adopting a TM and establishing an S-box algorithm generated from the 2D HM; The Lyapunov exponents and bifurcation diagrams of the chaotic dynamic behaviors for the S-box; PRBG was constructed based on with the DIFF approach; SCR, XOR, and PER operations; SECEVAMs: InE, HA, ACC, UACI, NPCR, KSP, KSS, and TCA; Good encryption efficiency; Against differential and statistical various attacks.
Xu et al. [33]	Lightweight MIE required more secure and efficient encryption algorithm; The PRCFM with 1D SAM chaotic map for the SCR-DIFF CRY approach to resist plaintext attack; The 1D SACM had a larger chaotic range and enhanced dynamic complexity; PER and SHA-256 operations; SECEVAMs: KSP, MSE, PSNR, HA, ACC, InE, NPCR, UACI, KSS, and TCA; Acceptable performance in both encryption time and robust security.
Hazzazi et al. [34]	The TMCP was designed to generate PRBG as BC encryption; The S-box and XOR operation were combined into the BC encryption enhancing security and increasing the strengths of TMCP; The BC encryption involved block-wise SUB and PER; SECEVAMs: HOA, energy, contrast, ACC, InE, MSE, RMSE, PSNR, AD, MD, STC, NCC, NAE, NPCR, UACI, and BACI; The MIE could combat statistical attacks, and had better accurate and security.
Zhuang et al. [35]	The new 5DMBMWCM; 5DMBMWCM with a relatively large Lyapunov exponent; QR decomposition, XOR, and bit-level SCR operations; SECEVAMs: InE, KSP, HA, KSS, NPCR, UACI, ACC; The KSP of the MIE was relatively large; The KSS was relatively strong; It better resisted of statistical attacks for MIs.
Lin et al. [36]	The chaos-based JPEG2000 MI CRY; JPEG2000 was a wavelet (TRAN)-based MI format; 2D chaotic LM phase SCR, a chaotic PER, and single-layer chaos-based MI pixels SCAN operations; SECEVAMs: VA, NCC, and PSNR.

**Table 3:** Technical notes on chaos-based MIE using the ROI, DNA, and AI schemes.

References	Technical Notes
Dua et al. [37]	An advanced IE algorithm using an ICFCM with a wider and more uniform chaotic range, a more positive Lyapunov exponent, and non-blank and non-stable windows; Three intermediate keys are produced; Three chaotic sequences are generated using the novel ICFCM, ToCCM, and CPM; DNA and DIFF operations; The features of DNA operations include huge storage, low power requirements, and extreme parallelism; SECEVAMs: PC, ACC, KSP, BCR, MSE, PSNR, TCA, GLIE, NPCR, and UACI.
Zhang et al. [38]	MIs can be divided into the ROI, and ROB; ROI is the part of an MI with diagnostic information; ROB is the part without visual or medical significance; 2D chaotic MLM, SHA-256 HF, ROI, ROB, PER, and SUB operations were integrated into the semi-full CRY mode; PER operation was used to encrypt the ROI, and the SUB operation was used to encrypt the entire image; 3D/4D chaotic MLM, SHA-256 HF, PER, and SUB operations were integrated into the full CRY mode; CONF, which was used to change the pixel positions in MIs; DIFF, which is used to change the value of pixels in MIs; SECEVAMs: GLIE, NCC, KSS, KSP, NPCR, UACI, and TCA; Semi-full CRY mode exhibited good security performance and a lower time complexity; Full CRY mode exhibited better security performance and a higher time complexity.
Zhang et al. [39]	ROI within massive MIs; ROI and ROB were segmented from the MIs; ROIs of the massive MIs were encrypted using Lorenz hyperchaotic maps; HM, XOR, SCR and DIFF operations; SECEVAMs: HA, VARH, GLIE, PC, ACC, NPCR, UACI, SSIM, KSP, KSS, and TCA; Chaos-based massive MIE technology used 13 parameters in the encryption approach; Calculation accuracy of the 64 bit double precision data was $10^{-15}$; KSP value of the massive MIE was ${\left(10^{15}\right)}^{13}=10^{195};$ Higher security and encryption efficiency.
Chen et al. [40]	Chaotic CRY systems exhibit the encryption characteristics of unpredictability, pseudorandomness, and initial value sensitivity; 1D chaotic maps, such as LM and SM, have small parameter ranges and limited chaotic behavior; MDCMs have long generation times and are expensive; A bifurcation diagram and the Lyapunov exponent of the LSM showed that it has good CRY characteristics; LSM has a shorter operation time complexity, larger encryption parameter range, better randomness, and better KSP; Clinical images had a high correlation between the image pixels and the centralized distribution of the clinical image information; Intra, and inter-block SCR schemes were adopted to encrypt the ROI and entire image, respectively; Hash operation of the SHA-512 HF, and encryption effects; SECEVAMs: KSP, KSS, HA, PC, ACC, GLIE, NPCR, UACI, HU, and TCA; High Enc efficiency, good practicability, and resistance to attack.
Kiran et al. [41]	Low computational complexity technologies; BC-based ROI and ROB operations; Significant ROI was encrypted using the PER operation; DM and XOR operations were used to achieve the DIFF function for the PER-based ROI; ROB was not encrypted; SECEVAMs: HA, GLIE, MSE, NPCR, UACI, SSIM, BER, NCC, PSNR, KSP, and TCA.
Demirkol et al. [42]	New nonlinear MCC, DNA coding scheme, XOR operation, and DIFF-CONF method to design an RT MIE system for application to the healthcare industry; Sufficient security; Chaos-based dynamic behavior using the demonstration of phase portraits, Lyapunov exponents, and bifurcation diagrams; The main advantages related to the use of the DNA coding scheme include a large KSP, and low computational power; SECEVAMs:TCA, HA, UACI, NPCR, and GLIE; Resistance against various attacks, and the high plaintext sensitivity.
Wenzheng et al. [43]	DICOM MI had a large volume and high computational complexity; 3D SCCLM provided the complexity of chaotic sequences and enhanced the robust function of MIE; Double-layer random DNA encoding DIFF process, fractal random PER process, and SHA-256 operation; SECEVAMs: HA, MSE, PSNR, SSIM, ACC, InE, KSP, NPCR, UACI, and KSS; The CRY algorithm had better performance in solving plaintext, statistical, and differential attacks.
Chen et al. [44]	Selective region MIE approach based on the CCCM; The CRY system consists of two stages: ROI CRY and full MIE; CCCM has better KSP, and fast computation and low complexity of low-dimensional chaotic characteristics; The modified2D Joseph traversal CONF mechanism is combined to SCR the positions of pixels in each sub-block, and further changed their pixel values with a round of DIFF; The block V-shape SCR scheme was used to SCR the entire image, and SHA-512 HF was integrated into the CCCM-based MIE system; SECEVAMs: KSP, KSS, InE, local InE, HA, ACC, NPCR, and UACI.
Chen et al. [45]	ROI-based selective lightweight MI cipher was proposed using PLM limiting hackers practical use of smart devices; The characteristics of smart devices were limited memory, low power, and low computing capability; PER-DIFF, XOR, bit-level circular shifts, and pixel shuffling operations; SECEVAMs: ACC, GLIE, KSP, NPCR, UACI, TCA, and PSNR.
Xu et al. [46]	Multiple MIs of arbitrary quantities and sizes; Enhanced security chaos-based TLCMCML; Innovation TLCMCML has better chaotic features, high randomness, high unpredictability, ergodicity, and a larger chaotic range; The odd-even interleaving PER scheme was adopted to individually SCR the ROI in each MI; A synchronous SCR, SHA-512 HF operation, bit-level PER-DIFF method; SECEVAMs: KSP, KSS, GLIE, ACC, NPCR, UACI, and TCA; Excellent CRY effectiveness and provides high-security MIE technologies.
Pham et al. [47]	TFWBCC-based decryption approaches; 4D HCM, and XOR operations; A highly robust, and precise secure MIE algorithm; Excellent HA, GLIE, and PSNR performance metrics.
Li et al. [48]	Chaos-based AI MIE; Privacy protection; High-dimensional features were adaptively extracted from the original MI using ResNet; More flexible and secure CRY model; Chaotic LM, and XOR-SCR operations; SECEVAMs: ACC, InE, and HA; The chaotic ResNet-LM MIE system can achieve higher security and RT encryption.

**Table 4:** Technical notes on chaos-based MIE applied to medical cloud, IoMT, mHealth, healthcare, and telemedicine.

References	Technical Notes
Chen et al. [49]	MIs are widely used in modern clinical diagnosis and treatment; MIs containing confidential information relating to patient’ privacy; Stored and transmitted using cloud-based networks; SCR is performed on plain images, and a cipher image is generated; An adaptive MIE algorithm based on LSM chaotic mapping to increase the robustness; SECEVAMs: HA, KSP, KSS, GLIE, NPCR, UACI, and NCC; Effectively protects against attacks, with better security and robustness.
Parvees et al. [50]	PNG and LCG were used with a 2D LCCM to protect the patient confidentiality of DICOM images during storage and transmission on a cloud-based platform; XOR, CONF, DIFF, pixel MAS, and SCR encryption operations; SECEVAMs: KSS, HA, PC, GLIE, NPCR, and UACI.
Vasegh et al. [51]	A synchronization chaotic approach with a fast-reaching finite time was de signed in the transceiver, as an encrypted key generator for a FORKCS; An XOR operation was performed to protect the MIs of patients; SECEVAMs: HA, VARH, PC, GLIE, NPCR, UACI, and TCA; Security and robustness of the chaos-based MIE.
Bhattacharjee et al. [52]	Storage and transmission of digital MIs for detecting and treating diseases increased significantly in hospitals during the COVID-19 pandemic; 1D LM; MLM was developed to increase unpredictability and encryption robustness; A variable-length gray-level code, and XOR operation are integrated into the generation of the encrypted key to CONF the intruder and subsequently used as the initial parameters of both MLMs; One-stage image pixels undergo PER using the address code that is generated from the sorting TRAN of the first MLM; A complete DIFF procedure is applied to the entire image using the second MLM to overcome differential and statistical attacks; SECEVAMs: PC, ACC, NPCR, UACI, KSS, and KSP; High-encryption performance and robust resistance to various attacks.
Jan et al. [53]	Highly efficient and resilient SEC for smart telehealth; IF framework based Hessenberg TRAN and chaotic encryption; Protects security and decreases the computational time; IEFHAC algorithm comprises three operations: XOR, ROT, and PRBGs; LM and SM were integrated into IEFHAC to achieve efficient CONF and the Hessenberg household transform was used to achieve DIFF efficiency; SECEVAMs: HA, CSOT, NPCR, UACI, KSP, KSS, SSIM, NCC, BER. and TCA; Computationally efficient solutions.
Sarosh et al. [54]	SEC of MIs is an important requirement in telemedicine, and cloud-based healthcare systems; Protecting the encryption and confidentiality of MIs transmitted through the cloud-based medicine system; 3D chaotic PLM was adopted to generate a PNG keystream, which was used to perform 8-bit (pixel) and 2-bit DNA and PER operations on MIs; XOR and DIFF operations are integrated into the chaos-based MIE to enhance security; SECEVAMs: HA, VARH, NCC, PSNR, NPCR, UACI, and TCA.
Sujarani et al. [55]	Disease diagnosis; Protecting medical clinical images is a significant design challenge in e-healthcare and telemedicine with RT application; Novel dynamic circular bit shifting, and bit-flipping methods in the CONF-DIFF phase of the MIs provided better security and confidentiality; 3D chaos-based HYM is utilized to generate three chaotic sequences with XOR operation; Robustness, higher randomness, high efficiency, higher complexity, unpredictable behavior, larger KSP, and KSS; SECEVAMs: GLIE, KSP, KSS, ACC, MSE, NPCR, UACI, SSIM, FSIM, NAE, PSNR, MD, and STC.
Chidambaram et al. [56]	Massive MIE plays a crucial role in the secure sharing, storing, and transmission of MIs; ROI and non-ROI of MI design aspects; Random pixel indices of the non-ROI of MI; 1D TM; A 3D LHM was utilized to the CONF encryption process of ROI-MI; Assuming each encryption key parameters has 14-digit precision, the 3D LHM has six key parameters; therefore, the 3D LHM provides a widely KSP of $10^{14\times 6}.$ DNA-encoding DIFF process and XOR operation were used into the ROI-MI encryption mechanism; SECEVAMs: KSP, HA, ACC, InE, MSE, PSNR, SNR, MDE, ID, and NPCR; Robust MIE framework can be applied to the fields of HER, DICOM, cloud-based mHealth, and telemedicine system.
Yousef et al. [57]	COVIDOA-based chaotic CM and LM; CONF and DIFF; High security and computational efficiency; IoMT-based healthcare system; SECEVAMs: HA, InE, ACC, UACI, NPCR, MSE, and PSNR.
Ningthoukhongjam et al. [58]	In the era of mHealth and telemedicine, X-ray MIs were encrypted using public-key CRY; ECC and BGC; Superior encryption time performance; Small key length and fast encryption transactions; BCG encryption methodology was designed through BBS PRBG for the XOR–based MI stream cipher; Characteristics of ECC and BCC were unpredictability, and fortifying CRY issues; Pixel randomization of X–ary MIs was adopted through a JSMPCM; LM and QM; JSMPCM had a large KSP; SECEVAMs: InE, ACC, NPCR, UACI, and TCA.
Le et al. [59]	Sensitive patient information and reliable and efficient MIE was required for telemedicine; Simultaneous PER and DIFF framework (SPDF) was developed using a 1D CICM, and a modified Josephus traversing method; CPM and CICM were integrated into the 1D CICM, and it had higher unpredictability and ergodicity; Dynamic SCR scheme was adopted to the modified Josephus traversing method enhancing PER operation; SECEVAMs: KSP, HA, ACC, NPCR, UACI, InE, PSNR, SSIM, and TCA; MIE had high efficiency and could withstand chosen plaintex, and noise at tacks.
Man et al. [60]	A dynamic rotation MIE based on ILOCM; Solving the security problems for the digital MIs in IoMT transmission; ILOCM included new nonlinear adjustment terms and encryption parameters; ILOCM had a wider range of chaotic parameters and better chaotic characteristics, and the ILOCM-based PRBG were more unpredictable; ILOCM-based key generator was applied to the MIE scheme to achieve dynamic rotation of PER and double SCR–DIFF; SECEVAMs: KSP, MSE, InE, HA, ACC, PSNR, SSIM, NPCR, UACI, and TCA.
Wang et al. [61]	New dynamic BC of MIE technology using the TDCM; PACS in the hospital; XOR operations were adopted for the BC, and ACCs were analyzed to illustrate the effectiveness and feasibility of the TDCM-based cipher.
Ali et al. [62]	Chaos-based symmetric MI ciphers were designed using TLTM and HM for an e-healthcare platform; PER and DIFF operations were adopted for pixel SCR; SECEVAMs: KSP, KSS, ACC, HA, GLIE, NPCR, UACI, PSNR, MSE, and TCA; TLTM–based MIE is efficient and robust.
Ye et al. [63]	Watermarking and encryption security method; Smart healthcare systems; 4D HCM, 2D-HSM, Scrambling, PER–DIFF, and XOR operations; MI stream cipher; SECEVAMs: NPCR, UACI, PSNR, MSE, GLIE, and TCA.
Khurana et al. [64]	1D CSM; Better complex behavior, better randomness in nature, and larger chaotic range; MIE system for healthcare; MIs such as X-rays, MRI scans, CT scans, and UIs were protected by integrating PERT, DIFF, and XOR operations; SECEVAMs: NPCR, UACI, ACC, GLIE, KSS, BCR, MSE, and PSNR; Chaos-based encryption methods were used to maintain security of MIs, and ensure that only authorized users could access sensitive MIs.
Abirami et al. [65]	DICOM MIs were widely used in the fields of medical diagnostics processes, telemedicine, and healthcare, and online DICOM MIE methods were required; Effective DICOM MIE approaches using XOR operations with a secret process derived from an LHM, and high-security multi-DICOM MI access could be verified; SECEVAMs: MSE, SNR, PSNR, ACC, and Q-value.
Neela et al. [66]	BCDGE technology; Advanced levels of security overcoming hacking of IoMT-based MIs; Blockchain scheme, and chaotic deep generative adversarial network based CRY technology were adopted to enhanced MI security; Generated private key had larger KSP, excellent randomness, complexity, and high entropy. SHA-1 hash algorithm, XOR, and SCR–DIFF operations; SECEVAMs: KSP, InE, HA, UACI, NPCR, SSIM, MSE, ACC, and TCA.
Shahid et al. [67]	Primary concern related to MI is the privacy and security approaches with cloud-based transactions and safe web; Distributed and immutable feature of blockchain infrastructure was provided using chaotic TM MI cipher resistant brute-force attacks; Three encryption key parameters were adopted in the chaotic TM; It had larger KSP, KSS, high unpredictability, and high complexity; SM, XOR, PER, SHA-256, and scattered operations; SECEVAMs: HA, SIE, NPCR, UACI, MSE, PSNR, KSP, KSS, CDR, and CC.
Archana et al. [68]	Block chain scheme was adopted to decentralized the storage and transmission of encrypted MIs through the network; Block chain framework created accountability and controlled across all independent smart devices; Secured management at the IoMT-edge system; MIs were decomposed into R, G, and B in which these sub-images were SCR using MSM, HM, and ACM, respectively; MSM had more intricate dynamics behavior, and generated highly unpredictable chaos-based time sequences; SECEVAMs: NPCR, UACI, ACC, InE, and TCA.

**Table 5:** Important technical features and effectiveness of chaos-based MIE.

Technical Features and Effectiveness	References
AI, fuzzy, and NN	[6,47,48,66]
ASK	[6,22]
AU	[64]
BC	[2,5,6,22,34,41,61]
Block chain	[66,67,68]
Cloud	[49,50,54,56,67]
CONF	[4,8,21,22,28,30,32,38,42,44,50,52,53,55,56,57]
CT	[17,18,20,21,27,29,35,38,42,44,45,62,64,68]
DICOM	[19,27,28,43,50,56,65]
DIFF	[4,8,19,20,21,22,23,25,26,28,32,37,38,39,41,42,43,44,45,46,50,52,53,54,55,56,57,59,60,62,63,64,66]
Discard	[12,13,14]
DNA	[30,37,42,43,54,56]
EPR	[27,56]
Healthcare	[3,54,55,57,62,63,64,65]
HF	[15,23,25,33,38,40,43,44,46,66,67]
IoMT	[57,60,66,68]
LCG	[50]
Lightweight	[28,33,45]
LFSR and NLFSR	[24,27]
MAS	[1,19,21,38,50]
MRI	[17,19,21,22,25,27,28,29,30,31,35,38,39,42,45,64,68]
Mobile	[13,56,58]
PACS	[61]
PER	[6,12,13,14,17,18,19,22,23,25,26,28,29,32,33,34,36,38,41,43,45,46,52,54,59,60,62,63,67]
PET	[38,42]
PERT	[17,20,64]
PNG	[5,6,18,20,21,24,28,29,30,50,54]
PRBG	[32,34,53]
ROB	[38,39,41]
ROI	[38,39,40,41,44,45,46,56]
ROT	[53,60]
RT	[7,21,32,42,55]
SBOX	[21,32,34]
SC	[6,22,31]
SCR	[1,3,12,14,15,18,19,20,24,31,32,35,36,38,39,40,44,46,48,49,50,59,60,62,63,66,68]
SEC	[3,13,42,53,54]
SK	[5,6,22,25]
Storage	[3,49,50,52,56,68]
Telemedicine/telehealth	[3,53,54,55,56,58,59,65]
Transmission	[3,49,50,52,56,60,67,68]
SUB	[6,17,18,19,21,22,28,29,30,34,38,40]
SWA	[19]
TRAN	[6,26,36,52,53]
Ultrasound	[17,18,38,64]
X-rays	[17,18,21,27,28,30,32,33,34,35,36,38,42,45,52,53,58,62,64]
XOR	[2,6,14,18,20,21,27,28,29,30,32,34,35,39,41,42,45,47,48,50,51,52,53,54,55,56,58,61,63,64,65,66,67]

**Table 6:** Important chaotic maps for chaos-based MIE.

Chaotic Maps	References
2D HM	[32]
2D HSM	[63]
2D LM	[18]
2D LCCM	[50]
2D MLM	[38]
3D CM	[22]
3D HYM	[55]
3D LHM	[56]
3D MLM	[38]
3D PLM	[54]
4D HCM	[63]
4D MCS	[25]
4D MLM	[38]
ACM	[6,22,24,68]
BCDGE	[66]
BM	[18,21]
CHCM	[23,28]
CICM	[59]
CM	[57]
CCCM	[44]
CPM	[8,31,37,59]
CSM	[7,64]
CUB	[21]
ECEM	[19]
FORKCS	[51]
HM	[6,18,21,40,62,68]
HCM	[47]
HSCM	[20]
ICFCM	[37]
ILOCM	[60]
JSMPCM	[58]
LM	[2,6,8,12,13,14,15,27,29,30,31,36,40,48,52,53,57,58]
LCCM	[49]
LHM	[65]
LSM	[7,8,26,40,49]
LTM	[24,26]
MCC	[42]
MDCM	[40]
MBMWCM	[35]
MLM	[38,52]
MSM	[68]
PLM	[6,45]
QM	[58]
SCCLM	[43]
SM	[40,53,67]
SACM	[33]
TDCM	[61]
TLCMCML	[46]
TLTM	[62]
TM	[6,8,30,32,56,67]
TMCM	[34]
TOCCM	[37]

**Table 7:** Important SECEVAMs for chaos-based MIE.

SECEVAMs	References
ACC	[7,26,28,32,33,34,35,37,39,40,43,44,45,46,48,52,55,56,57,58,59,60,61,62,64,65,66,68]
AD	[34]
BACI	[34]
BCR	[37,64]
BER	[13,53]
CC	[67]
CDR	[67]
CSQT	[23,26,53]
EDR	[41]
Energy contrast	[34]
FSIM	[55]
GLIE	[6,7,8,17,18,19,22,23,24,25,27,28,29,30,37,38,39,40,41,42,45,46,47,49,50,51,55,62,63,64]
HA	[6,7,8,18,19,21,22,23,25,27,28,29,30,32,33,35,39,40,41,42,43,44,47,48,49,50,51,53,54,56,57,59,60,62,66,67]
HOA	[34]
HU	[40]
ID	[56]
InE	[32,33,34,35,43,44,48,56,57,58,59,60,66,68]
KSP	[6,7,8,18,19,20,21,22,23,25,26,27,29,30,32,33,35,37,38,39,40,41,43,44,45,46,49,52,53,55,56,58,59,60,62,66,67]
KSS	[6,7,8,18,19,20,21,22,23,24,25,26,27,29,30,31,32,33,35,38,39,40,43,44,46,49,50,52,53,55,62,64,67]
Local InE	[44]
MAE	[22,26]
MD	[34,55]
MDE	[56]
MSE	[15,17,22,26,28,29,33,34,37,41,43,55,56,57,60,62,63,64,65,66,67]
NAE	[34,55]
NCC	[12,13,17,18,20,27,29,30,34,36,38,41,49,53,54]
NPCR	[6,7,8,17,18,19,20,21,23,24,25,26,27,28,29,30,31,32,33,34,35,37,38,39,40,41,42,43,44,45,46,49,50,51,52,53,54,55,56,57,58,59,60,62,63,64,66,67,68]
PC	[6,7,8,19,21,22,23,24,25,26,30,37,39,40,50,51,52]
PCC	[15]
PRD	[12,13,14]
PSNR	[17,18,22,23,24,27,28,29,30,33,34,36,37,41,43,45,47,54,55,56,57,59,60,62,63,64,65,67]
RMSE	[22,27,34]
STC	[55]
SIE	[67]
SSIM	[24,27,39,41,43,53,55,59,60,66]
SNR	[56,65]
STC	[34]
STE	[67]
Q-value	[65]
TCA	[7,14,17,21,24,28,29,32,33,37,38,39,40,41,42,45,46,51,53,54,58,59,60,62,63,66,68]
UACI	[6,7,8,17,18,19,20,21,23,24,25,26,27,28,29,30,32,33,34,35,37,38,39,40,41,42,43,44,45,46,49,50,51,52,53,54,55,57,58,59,60,62,63,64,66,67,68]
UIQI	[22]
VA	[12,13,15,26,31,36]
VARH	[20,24,25,39,51,54]

## Abbreviations

1D	One dimensional
2D	Two-dimensional
3D	Three-dimensional
4D	Four-dimensional
4D MCS	Four-dimensional memristive chaotic sequence
5D	Five-dimensional
4O	Fourth-order
3D CM	Three-dimensional chaotic maps (SM, LM, and cubic maps)
ACC	Adjacent correlation coefficient
ACM	Arnold cat map
AD	Average difference
AES	Advanced encryption standard
AI	Artificial intelligence
ASK	Asymmetric key
AU	Authenticate
BACI	Blocked average changing intensity
BBS	Blum Blum Shub
BC	Block cipher
BCR	Bit correct ratio
BCDGE	Block chain based chaotic deep generative adversarial network encryption
BGC	Blum-Goldwasser cryptosystem
BM	Baker map
BER	Bit error rate
CC	Complexity of computation
CCCM	Cosine-cubic cascaded map
CDR	Ciphertext difference rate
CONF	Confusion
CHCM	Chen-based hyperchaotic map
CICM	Chebyshev iterative chaotic map
CPM	Chebyshev polynomial map
CT	Computed tomography
CRY	Cryptography
CM	Cosine map
CSM	Cosine -sine map
CSQT	Chi-square test
CUM	Cubic map
DE	Data encryption
DES	Data encryption standard
DICOM	Digital imaging and communication in medicine
DIFF	Diffusion
DM	Duffing map
DNA	Deoxyribonucleic acid
ECC	Elliptic curve crypographic
ECEM	Enhanced chaos-based economic map
ECG	Electrocardiography
EDR	Edge differential rate
EEG	Electroencephalography
EMD	Empirical mode decomposition
EPR	Electronic patient report
FSIM	Feature-similarity
FORKCS	Fourth-order Runge–Kutta chaotic system
GLIE	Global and local information entropy
HA	Histogram analysis
HCM	Hyperchaotic map
HDV	Histogram deviation value
HF	Hash function
HM	Henon map
HOA	Homogeneity analysis
HSM	Henon-sine map
HSCM	Hyperbolic sine chaotic map
HU	Histogram uniformity
HYM	Hyperbolic map
ICFCM	Improved cosine fractional chaotic map
ID	Irregular deviation
IE	Image encryption
ILOCM	Improved Lorenz chaotic map
IMF	Intrinsic mode function
IoMT	Internet of Medical Things
InE	Information entropy
JSMPCM	JoanS-MuraliP’s chaotic map
KSP	Key space
KSS	Key sensitivity
LCCM	Logistic coupled chaotic map
LCG	Linear congruential generator
LFSR	Linear feedback shift register
LHM	Lorenz hyperchaotic map
LM	Logistic map
LSM	Logistic sinusoidal map
LTM	Logistic-tent map
MAE	Mean absolute error
MAS	Masking
MBMWCM	Multi-band multi-wing chaotic map
MCC	Memristor-based chaotic circuit
MCM	Memristive chaotic map
MD	Maximum difference
MDE	Maximum deviation
MI	Medical image
MIE	Medical image encryption
MLM	Modified logistic map
MSE	Mean square error
MSM	Multi-scroll maps
MRI	Magnetic resonance imaging
NAE	Normalized absolute error
NCC	Normalized correlation coefficient
NLFSR	Nonlinear feedback shift register
NM	Nested map
NN	Neural network
NPCR	Number of changing pixel rate
PACS	Picture archiving and communication systems
PC	Pixel correlation
PCC	Person correlation’s coefficient
PER	Permutation
PERT	Perturbation
PET	Positron emission tomography
PLM	Piecewise linear map
PNG	Pseudorandom number generator
PRBG	Pseudorandom bit generator
PRCFM	Plaintext-related and ciphertext feedback mechanism
PRD	Percent root-mean-square difference
PSNR	Peak signal-to-noise ratio
QM	Quadratic map
QR	Quick response
RMSE	Root mean square error
ROB	Region of background
ROI	Region of interest
ROT	Rotated
RRCS	Runge–Kutta chaotic map
RSA	Rivest-Shamir-Adleman
RT	Real-time
SACM	Sine arcsine chaotic map
SC	Stream cipher
SCCLM	Spatiotemporal coupled chaotic lattice map
SCR	Scramble
SEC	Security communication
SECEVAM	Security evaluation method
SHA	Secure hash algorithm
SID	Statistical irregular deviation
SK	Symmetric keys
SLMM	Sine logistic modulation map
SM	Sine map
SNR	Signa-to-noise ratio
SPDF	Simultaneous PER and DIFF framework
SPO2	Oxygen saturation
SSIM	Structural similarity index measurement
STC	Structural content
SUB	Substitution
SWA	Swapping
TCA	Time complexity analysis
TDCM	Time-delay chaotic map
TLCMCML	Tent-logistic cross-mixed coupled map lattice
TLTM	Tent logistic tent map
TM	Tent map
TMCM	Tent-May chaotic map
ToCCM	Tangent over cosine map
TRAN	Transform
TSKFWBCC	Takagi-Sugeno-Kang Fuzzy Wavelet Brain Cerebral Controller
UIQI	Universal image quality index
UACI	Unified averaged changed intensity
UI	Ultrasound imaging
VA	Visual analysis
VARH	Variance of histograms
XOR	Exclusive or
